# Phosphoenolpyruvate Carboxykinase, a Key Enzyme That Controls Blood Glucose, Is a Target of Retinoic Acid Receptor-Related Orphan Receptor α

**DOI:** 10.1371/journal.pone.0137955

**Published:** 2015-09-18

**Authors:** Hiroshi Matsuoka, Akiho Shima, Daisuke Kuramoto, Daisuke Kikumoto, Takashi Matsui, Akihiro Michihara

**Affiliations:** Laboratory of Genome Function and Pathophysiology, Faculty of Pharmacy and Pharmaceutical Science, Fukuyama University, Fukuyama, Hiroshima, Japan; University of Hong Kong, HONG KONG

## Abstract

Phosphoenolpyruvate carboxykinase (PEPCK) catalyzes a committed and rate-limiting step in hepatic gluconeogenesis, and its activity is tightly regulated to maintain blood glucose levels within normal limits. PEPCK activity is primarily regulated through hormonal control of gene transcription. Transcription is additionally regulated via a cAMP response unit, which includes a cAMP response element and four binding sites for CCAAT/enhancer-binding protein (C/EBP). Notably, the cAMP response unit also contains a putative response element for retinoic acid receptor-related orphan receptor α (RORα). In this paper, we characterize the effect of the RORα response element on cAMP-induced transcription. Electrophoresis mobility shift assay indicates that RORα binds this response element in a sequence-specific manner. Furthermore, luciferase reporter assays indicate that RORα interacts with C/EBP at the PEPCK promoter to synergistically enhance transcription. We also found that cAMP-induced transcription depends in part on RORα and its response element. In addition, we show that suppression of RORα by siRNA significantly decreased PEPCK transcription. Finally, we found that a RORα antagonist inhibits hepatic gluconeogenesis in an *in vitro* glucose production assay. Taken together, the data strongly suggest that PEPCK is a direct RORα target. These results define possible new roles for RORα in hepatic gluconeogenesis.

## Introduction

Phosphoenolpyruvate carboxykinase (PEPCK) catalyzes a committed and rate-limiting step in hepatic gluconeogenesis, and its activity is tightly regulated to maintain normal blood glucose levels. PEPCK activity is primarily modulated through hormonal control of transcription [1–3]. The PEPCK promoter contains several *cis*-elements that functionally cooperate to respond to the thyroid hormone, glucocorticoids, and cAMP [4]. The glucocorticoid response unit consists of elements between -467 and -300, which include two tandem binding sites for the glucocorticoid receptor [5]. On the other hand, the cAMP response unit consists of four binding sites for CCAAT/enhancer-binding protein (C/EBP), and a canonical cAMP response element that recruits both CREB and C/EBP [6–9].

**Fig 1:**
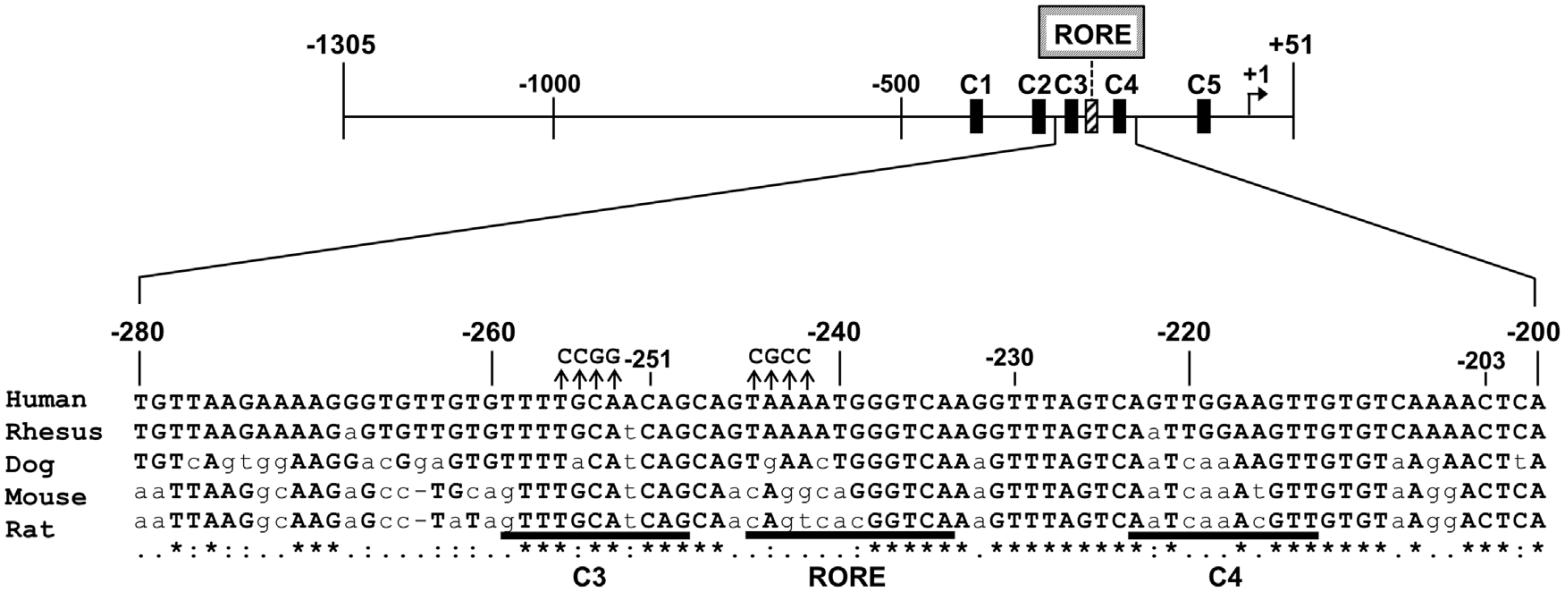
RORα and C/EBP binding sites in PEPCK. Schematic representation of RORα and C/EBP binding sites (RORE, C1, C2, C3, C4, and C5) in human PEPCK. The nucleic acid sequence is shown, with binding sites underlined and conserved sequences marked with asterisks. Mutations used in luciferase reporters are indicated with arrowheads. RORα and C/EBPβ regulate gene expression, mainly by binding their respective response elements.

C/EBP mediates the sensitivity of the PEPCK promoter to cAMP. Indeed, knockout of C/EBPβ decreases gluconeogenesis and circulating lipids [10]. Notably, a putative response element for retinoic acid receptor-related orphan receptor alpha (RORα) is found four bases downstream of a C/EBP site. This response element has a strongly conserved consensus sequence (Fig 1).

RORα regulates target gene expression mainly by binding as monomers to promoter response elements, which typically consist of a consensus AGGTCA half-site preceded by an A/T-rich sequence [11]. RORα-deficient mice that carry a natural deletion in the ligand-binding domain have cerebellar ataxia, a phenotype also observed in *Staggerer* (*sg*/*sg*) mutant mice [12], which express mutated RORα and additionally present vascular dysfunction, muscular defects, osteoporosis, immune abnormalities, and diet-induced atherosclerosis [13–15]. Although RORα is expressed in the liver, its contribution to hepatic function has not been appreciated until recently. In mice, loss of RORα affected expression of multiple hepatic phase I and phase II enzymes that metabolize drugs [16]. The receptor has also been shown to directly regulate the cytochrome P450 enzymes CYP7B1 [17] and CYP2C8 [18]. Moreover, the transcriptional activator SRC-2 functions as a coactivator with RORα to modulate expression of G6Pase [19], an essential, rate-limiting enzyme that controls glucose release into the plasma. Furthermore, RORα deficiency and treatment with RORα antagonists inhibit PEPCK expression and glucose production in mice [20, 21]. Similarly, overexpression of Rev-erbα, the physiological repressor of RORα, also suppresses expression of G6Pase and PEPCK in HepG2 cells. Conversely, silencing of Rev-erbα significantly stimulates G6Pase and PEPCK expression [22–24].

Nevertheless, the role of RORα in modulating PEPCK promoter activity is not clear. In this paper, we demonstrate that RORα cooperates with C/EBPβ to directly stimulate PEPCK expression.

## Methods

### Electrophoresis mobility shift assay

**Fig 2:**
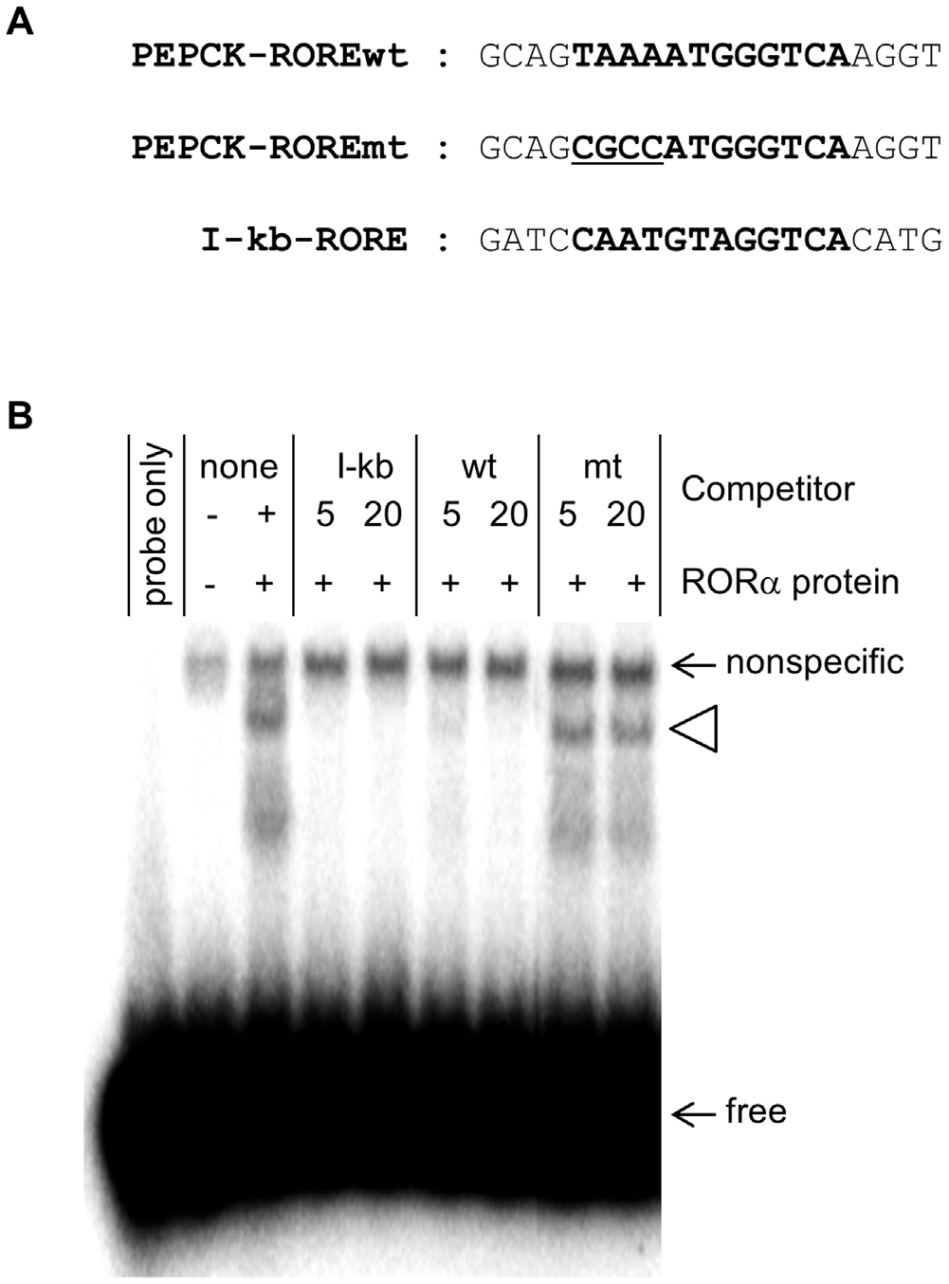
RORα binds to a putative response element in PEPCK. (A) Sequence of the putative RORα response element (in boldface) in human PEPCK. The probe mt is equivalent to this motif, except that underlined nucleotides have been mutated. The I-kb probe contains the known RORα response element in NF-kappaB inhibitor α. (B) EMSA was used to test the ability of unlabeled wild type and mutated probes, at 5- and 20-fold excess, to inhibit binding of RORα to the putative response element (open arrowhead). The positions of free probe (free) and nonspecific probe of RORα for crude protein (nonspecific) were indicated. All reactions except lane 1 contain crude products from *in vitro* translation. In lane 2, the probe was incubated with the crude product obtained from *in vitro* translation in the absence of the RORα expression vector.

RORα was obtained by *in vitro* translation using the *In Vitro* Transcription Coupled Rabbit Reticulocyte Lysate System (Promega, Madison, WI, USA), following the manufacturer’s protocol. The empty expression vector was used as control. Subsequently, the RORα crude product from *in vitro* translation was incubated with radiolabeled DNA probes for 20 min at 30°C in a reaction mixture that also contained 20 mM HEPES pH 7.0, 50 mM KCl, 1 mM dithiothreitol, 5% glycerol, 20 μg/mL dI/dC, and 0.025% NP-40. Electrophoretic mobility shift assay was then performed essentially as described previously [25]. The PEPCK probe was prepared by radiolabeling synthetic double-stranded DNA using [γ-32P] ATP (Perkin-Elmer, Waltham, MA, USA) and T4 polynucleotide kinase (Takara Bio, Shiga, Japan). Unlabeled probe was used as competitive inhibitor, along with a mutated probe and I-kb, which contains a known RORα response element [26] and was used as positive competitive control (Fig 2A). In this assay, 0.02 pmol ^32^P-radiolabeled wild type probe was incubated with RORα crude product from *in vitro* translation, along with 0.1 pmol and 0.4 pmol unlabeled DNA. Probe sequences are listed in S1 Table.

### Luciferase reporters

The human PEPCK promoter, from -1305 to +51 relative to the transcriptional start site, was amplified by PCR and inserted into the luciferase expression vector PGVB2 (Nippon Gene, Tokyo, Japan). This plasmid was then used in linker-scanning mutagenesis [27] to generate the deletion plasmids dC1 (deletion of C1 from -1305 to -323), dC2C3 (deletion of C2 and C3 from -323 to -251), dC4 (deletion of C4 from -251 to -203), and dC5 (deletion of C5 from -78 to -43). The mutated promoter ROREmt was generated by PCR using the wild type sequence as template. Briefly, the primers ROREmt-FW and ROREmt-RV were synthesized to incorporate the desired mutation (Fig 1 and S1 Table). These primers were then used in PCR reactions with PGVB2-FW and PGVB2-RV, respectively, to generate overlapping fragments that contain the mutation. The resulting fragments were then used in a final PCR reaction with PGVB2-FW and PGVB2-RV to generate a continuous mutated fragment, which was then cloned as a *Mul*I/*Hind*III-fragment into PGVB2. The mutated promoter C3mt was generated in a similar manner using the primers C3mt-FW and C3mt-RV to incorporate mutations in the C3 binding site (Fig 1 and S1 Table). Plasmids were purified using QIAGEN Plasmid Mini Kit (Qiagen, Valencia, CA, USA) according to the manufacturer’s protocol.

### Transfection and luciferase activity assay

**Fig 3:**
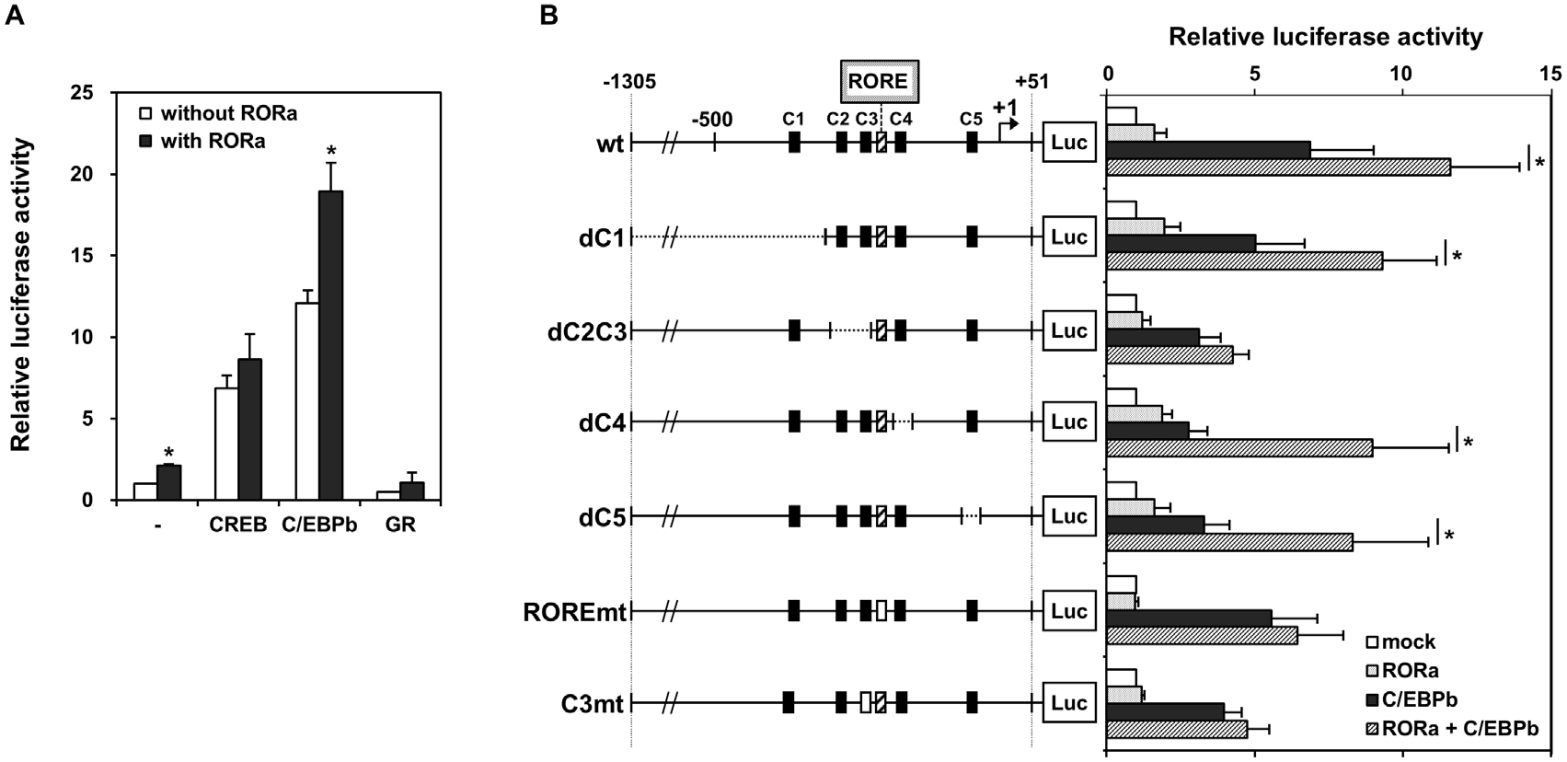
The PEPCK promoter is directly activated by C/EBPβ and coactivated by RORα. (A) Transactivation of the PEPCK promoter by the nuclear receptors CREB, C/EBPβ, and glucocorticoid receptor with or without RORα. HepG2 cells were transfected with luciferase under the control of the PEPCK promoter (-1305 to +51). Data are fold transactivation over basal activity, and are reported as mean ± S.E. (n = 3). (B) HepG2 cells was transfected with RORα (dotted bars), C/EBPβ (closed bars), or both (hatched bars), along with luciferase driven by wild type or mutated PEPCK promoters. Data are mean ± S.E. (n = 3). Mutations in the promoter include deletions of the C1, C4, and C5 binding sites, as well as deletion of both C2 and C3 and mutation of C3 and of the RORα response element. *, *p* < 0.05.

**Fig 4:**
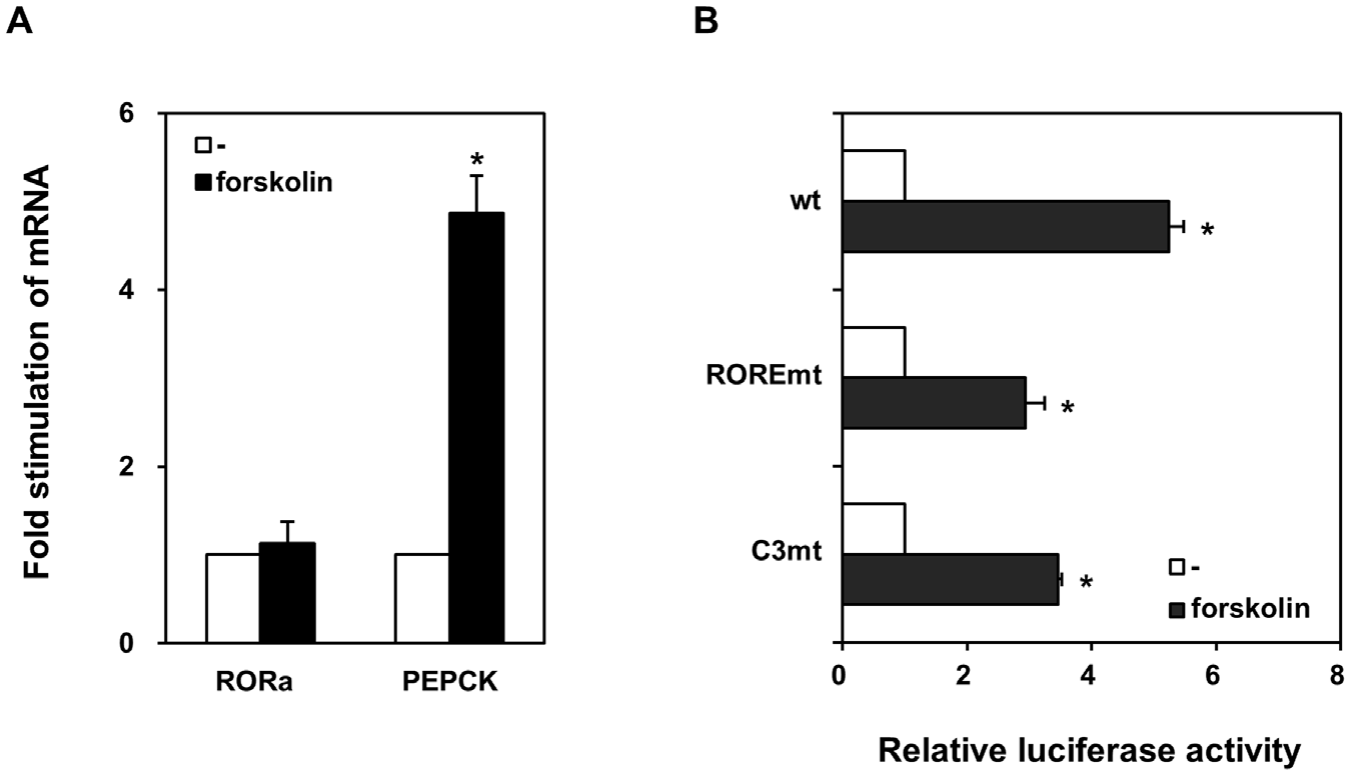
cAMP-dependent stimulation of the PEPCK promoter as mimicked by forskolin. (A) HepG2 cells were treated with (closed bars) or without 10 μM forskolin (open bars). Expression of RORα and PEPCK was analyzed by qRT-PCR, and normalized to 18S rRNA. Data are mean ± S.E. (n = 3). (B) HepG2 cells were transfected with luciferase under the control of wild type or mutated PEPCK promoters, and treated with (closed bars) or without 10 μM forskolin (open bars). Data are shown as mean ± S.E. of three experiments. *, *p* < 0.05.

HepG2 cells were transfected using Lipofectamine 2000 (Life Technologies, Gaithersburg, MD, USA) according to the manufacturer’s protocol. Briefly, cells were seeded at 1 × 10^5^ cells/well in Dulbecco’s modified Eagle’s medium containing 10% fetal calf serum. After 1 day at 37°C and 5% CO_2_, each well was transfected for 12–16 h with a mixture of 50 ng luciferase reporter plasmid, 100 ng nuclear receptor expression plasmid, 100 ng pUC19, and 50 ng β-galactosidase reporter plasmid, which was used to normalize luciferase activity. Cells were grown another 24–32 h in fresh media (Fig 3), and finally stimulated with 10 μM forskolin for 8 h (Fig 4B). Data were collected from at least four independent experiments.

### Quantitative reverse transcription-PCR (qRT-PCR)

HepG2 cells were seeded at 1 × 10^5^ cells/well in Dulbecco’s modified Eagle’s medium supplemented with 10% fetal calf serum. After 24 h at 37°C and 5% CO_2_, cells were stimulated with 10 μM forskolin (Sigma, St. Louis, MO, USA) for 8 h. Total RNA was then prepared using ISOGENE reagent (Wako Pure Chemical, Osaka, Japan), and reverse transcribed for 90 min at 37°C in a reaction containing 25 mM Tris-HCl pH 7.8, 37.5 mM KCl, 1.5 mM MgCl_2_, 5 pmol/μL oligo dT (Takara Bio), 0.5 mM dNTP (Applied Biosystems, Foster City, CA, USA), 10 mM dithiothreitol, 20 units RNase inhibitor (Takara Bio), and 200 units Moloney murine leukemia virus reverse transcriptase (MBI Fermentas, St Leon-Rot, Germany). Synthesized cDNA was then directly amplified using a Roche Light Cycler in a 15 μL reaction containing SYBR green Real-Time PCR master mix (Toyobo, Osaka, Japan) and 1 μM primers. RORα gene was amplified using the primers rtRORA-FW and rtRORA-RV in 40 cycles at 95°C for 10 s, 56°C for 10 s, and 72°C for 15 s following initial denaturation at 95°C for 2 min. PEPCK expression was quantified in a similar manner using rtPEPCK-FW and rtPEPCK-RV. β-actin, amplified using rtACTB-FW and rtACTB-RV, was used as internal control, along with 18S rRNA, which was amplified using rt18SrRNA-FW and rt18SrRNA-RV (S1 Table). Data were collected from at least three independent experiments.

### Suppression of endogenous RORα by siRNA

siRNAs that target different sequences in RORα (siRORa-258 and siRORa-1388) were generated by *in vitro* transcription T7 kit (Takara Bio). siRNA against green fluorescent protein was used as negative control (siGFP), along with a scrambled siRNA against RORα (siRORa-mt). siRNA sequences are listed in S2 Table. HepG2 cells were seeded in a 24-well plate at 1 × 10^5^ cells/well, and transfected with siRNA the following day. Cells were harvested 48 h after transfection, and total RNA was extracted. RORα and PEPCK were quantified by qRT-PCR as described.

### Glucose production assay

HepG2 cells were cultured in 12-well plates at 3 × 10^5^ cells/well, and allowed to attach for 24 h. Cultures were then treated for 4 h with and without 10 μM SR1001, a RORα ligand, in serum-free medium. Subsequently, cultures were washed twice with phosphate buffered saline to remove glucose, and incubated in glucose-free D-MEM containing 1 mM pyruvate and 10 mM lactate. Glucose concentration was measured 3 h later in 50 μL samples of the media using Glucose Assay Kit II (BioVision, Mountain View, CA, USA).

## Results

### Identification of a RORα response element in PEPCK

The ability of RORα to bind its putative response element in the PEPCK promoter was tested by EMSA. The location and sequence of this response element are shown in Figures (Figs 1 and 2A). Briefly, a 20-bp fragment spanning positions -250 to -230 of the PEPCK promoter was end-labeled, and incubated with RORα obtained by *in vitro* translation (Fig 2B). The receptor induced sequence-specific electrophoretic mobility shifts that were inhibited by addition of excess unlabeled probe such as I-kB, which contains a known RORα response element in NF-kappaB inhibitor α (Fig 2B). Notably, the mutated probe was unable to inhibit RORα binding to the putative response element (Fig 2B).

### C/EBPβ and RORα synergistically activate PEPCK expression

As both C/EBPβ and RORα can bind the cAMP response element in PEPCK, we investigated the effect of overexpressing both. In HepG2 cells, C/EBPβ alone strongly stimulates, by 12-fold, expression of luciferase under the control of the PEPCK promoter (Fig 3A). However, RORα by itself stimulates expression only 2-fold (Fig 3A). Notably, when both proteins were overexpressed, reporter activity increased about 20-fold (Fig 3A). Mutation of the downstream C/EBPβ binding site C1, C4 and C5 did not affect this synergy, suggesting that C/EBPβ synergizes with RORα at the RORα response element (Fig 3B). Other nuclear receptors, including CREB and glucocorticoid receptor, did not cooperate with RORα (Fig 3A).

To examine whether RORα and C/EBPβ synergistically stimulate transcription of PEPCK genes, both proteins were overexpressed in HepG2 cells along with luciferase under the control of wild type or mutated PEPCK promoters (Fig 3B). Mutations in the promoter include deletions of the C1, C4, and C5 binding sites, as well as deletion of both C2 and C3 and mutation of C3 and of the RORα response element. Synergy between RORα and C/EBPβ was markedly disrupted by deletion of C2 and C3, or point mutation of RORE and C3. Thus, mutation of the RORα response element abolished both the ability of the receptor to activate transcription and to synergize with C/EBPβ (Fig 3B). This mutation also diminished transactivation by C/EBPβ. Similar results were obtained when the C3 site was mutated (Fig 3B), or when C2 and C3 were both deleted. Deletion of C1, C4, and C5 did not significantly diminish PEPCK activation by RORα, but markedly reduced the cooperative effect of C/EBPβ (Fig 3B). These results suggest that each binding site is required for a synergistic response to C/EBPβ and RORα although they are similar and could theoretically compensate for the loss of each other.

### RORα response element and cAMP-dependent PEPCK expression

In HepG2 cultures, 10 μM forskolin mimics cAMP signaling and stimulates PEPCK transcription about 5-fold without affecting expression of RORα (Fig 4A). In a luciferase reporter assay, cAMP-dependent activation of PEPCK decreased by about 50% by mutation of C3 and of the RORα response element four bases downstream (Fig 4B). Thus, transcriptional activation by cAMP depends only modestly on these motifs.

### siRNA against RORα reduces PEPCK expression

**Fig 5:**
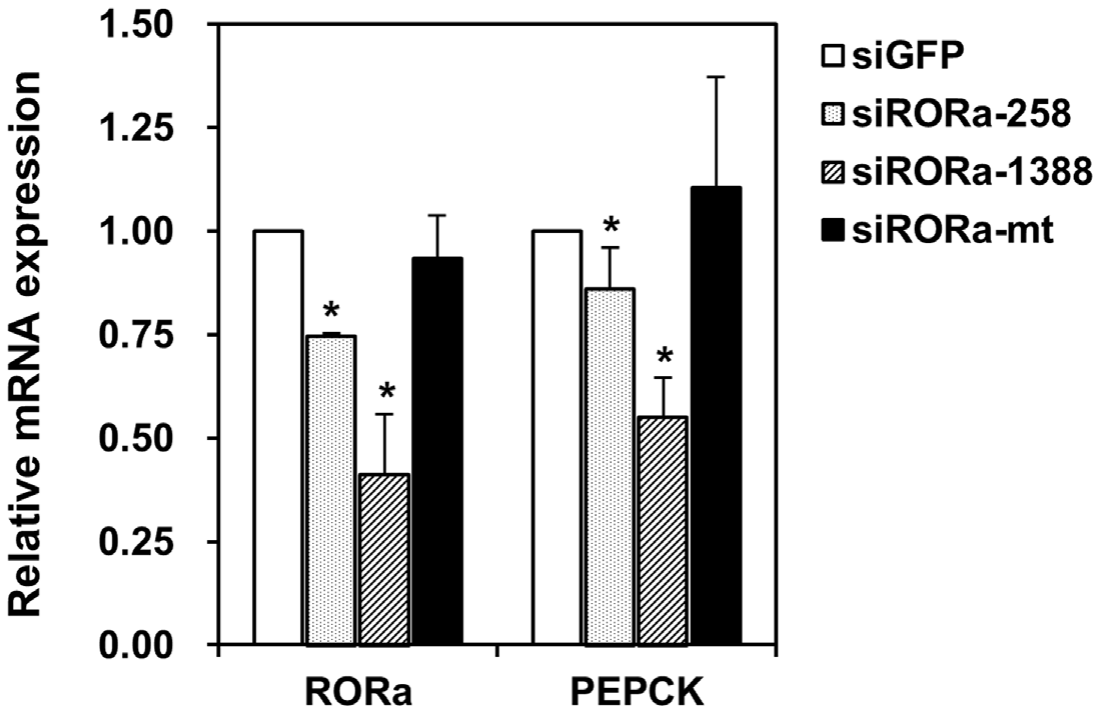
Effect of RORα deficiency on PEPCK expression. Suppression of endogenous RORα by siRNA significantly decreased PEPCK mRNA in HepG2 cells. HepG2 cells were transfected with 50 nM siRNA, and analyzed by qRT-PCR to measure expression of RORα and PEPCK. siRNAs that target sequences around 258 bp (siRORa-258, dotted bars) and 1388 bp (siRORa-1388, hatched bars) downstream of the RORα start codon decreased RORα and PEPCK mRNA levels, while a scrambled siRNA (siRORa-mt, closed bars) did not. siRNA against green fluorescent protein was used as negative control (siGFP, open bars). Data are mean ± S.E. of three experiments, and are normalized to 18S rRNA. *, *p* < 0.05.

While small interfering-RNA against green fluorescent protein did not affect transcription of PEPCK, targeted knockdown of RORα also decreased PEPCK transcription. Indeed, siRNA that target sequences around 258 bp and 1388 bp downstream of the RORα start codon suppressed PEPCK transcription by 86.0% and 55.2%, respectively, while a scrambled siRNA did not (Fig 5).

### A RORα antagonist reduces glucose production

**Fig 6:**
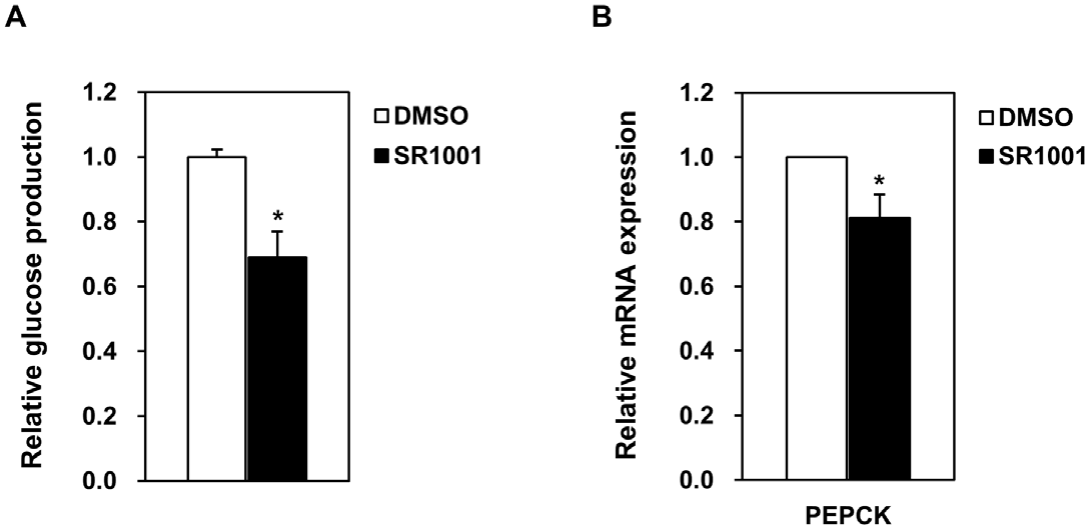
*In vitro* gluconeogenesis is suppressed by a RORα antagonist. (A) Glucose production in HepG2 cells incubated for 4 h in 10 μM SR1001, a RORα antagonist, or in DMSO. (B) Effect of 10 μM SR1001 on PEPCK mRNA expression in HepG2 cultures 7 h after treatment. Data are mean ± S.E. of triplicate experiments. *, *p* < 0.05.

In HepG2 cultures, 10 μM SR1001, a RORα antagonist, inhibits PEPCK transcription to 81.3% (Fig 6B). The antagonist also reduces hepatic gluconeogenesis to 68.8% of untreated cells (Fig 6A).

## Discussion

PEPCK catalyzes a committed and rate-limiting reaction in hepatic gluconeogenesis, and its activity is tightly regulated by hormones and cAMP to maintain normal blood glucose levels [1–3]. The cAMP response unit consists of five *cis*-elements, including four binding sites for C/EBPs, and a canonical cAMP response element for CREB. In particular, the C3 binding site is absolutely conserved among humans, rhesus monkeys, dogs, mice, and rats (Fig 1), suggesting that transcriptional activation of PEPCK by C/EBPβ [4] is a process common to mammals.

Notably, a search for RORα response elements in human genes identified PEPCK as a putative target. A typical RORα response element consists of a consensus AGGTCA half-site preceded by an A/T-rich region [11]. In PEPCK, the response element is four bases downstream of the C3 site, and is absolutely conserved in humans and rhesus monkeys, but not in dogs, mice, and rats (Fig 1). Using electrophoretic mobility shift assay, we demonstrated that RORα binds to this response element (Fig 2B). Furthermore, luciferase reporter assays indicate that the receptor modestly stimulates expression (Fig 3). In addition, we found that cAMP-induced stimulation of PEPCK expression depends on the RORα response element (Fig 4). Finally, we showed that suppression of RORα expression by siRNA also significantly decreased PEPCK transcription (Fig 5). Taken together, the data strongly indicate that PEPCK is a direct RORα target.

Consequently, an antagonist of RORα also inhibits PEPCK expression and hepatic glucose production (Fig 6). Conversely, knockdown or inhibition of Rev-erbα, a transcriptional regulator that competes with RORα for binding the RORα response element, enhances PEPCK abundance and glucose production [22–24]. Thus, it appears that hepatic gluconeogenesis *via* PEPCK and G6Pase depends crucially on the balance between RORα and Rev-erbα activity. Notably, transcription of the clock gene Bmal1 seems to depend on the same competitive balance [28, 29].

In addition, we found that RORα cooperates with C/EBPβ to synergistically stimulate PEPCK expression (Fig 3). Indeed, the PEPCK promoter has been previously shown to respond to several multisubunit complexes. For instance, glucocorticoid receptor was recently shown to interact with peroxisome proliferator activated receptor gamma at the PEPCK promoter [30]. Finally, the ability of the promoter to respond to cAMP is mediated by synergistic interaction between the cAMP response element and a complex regulatory element that recruits transcription factors enriched in the liver [31, 32].

RORα regulates target gene expression mainly by binding as monomers to promoter response elements. For example, the receptor has been shown to directly regulate G6Pase transcriptional activation by the SRC-2 activator [19]. The physiological significance of RORα has been widely characterized. For instance, overexpression of RORα in rat insulinoma cell lines increased expression and secretion of insulin [33]. Moreover, the homozygous staggerer (*sg*/*sg*) mouse, which expresses mutated RORα has improved insulin sensitivity and enhanced glucose uptake in skeletal muscle [34]. In addition, a RORα inverse agonist suppresses insulitis and prevents hyperglycemia in a mouse model of type 1 diabetes [35]. RORα also potentially regulates transcription of brain-derived neurotrophic factor, which is associated with Alzheimer’s disease [36–38]. Finally, IkappaBzeta cooperates with RORα to regulate the development of T helper 17 cells during inflammation [39–42].

In summary, our data suggest that human PEPCK is a direct target of RORα with a functional response element at positions -245 to -234. Our results define possible new roles for RORα in hepatic gluconeogenesis, possibly by regulating PEPCK expression. Finally, understanding the interaction between C/EBPβ and RORα may potentially help design specific drugs to treat inflammation, diabetes, and obesity.

## Supporting Information

S1 Table Primers. (PDF)

S2 Table siRNAs. (PDF)
