# Supplementary material for: Phosphoenolpyruvate Carboxykinase, a Key Enzyme That Controls Blood Glucose, Is a Target of Retinoic Acid Receptor-Related Orphan Receptor α
Source: PLoS One. 2015 Sep 18;10(9):e0137955. doi: 10.1371/journal.pone.0137955 (PMC4575163; doi:10.1371/journal.pone.0137955)
Supplement: S1 Table — (PDF) [file pone.0137955.s001.pdf]

S1 Table. Primers. Mutated sequences are underlined.

Primer	Sequence (5′–3′)
Electrophoresis mobility shift assay	
PEPCK-ROREwt-sense	GCAGTAAAATGGGTCAAGGT
PEPCK-ROREwt-antisense	ACCTTGACCCATTTTACTGC
PEPCK-ROREmt-sense	GCAGCGCCATGGGTCAAGGT
PEPCK-ROREmt-antisense	ACCTTGACCCATGGCGCTGC
Ikb-RORE-sense	GCAGCGCCATGGGTCAAGGT
Ikb-RORE-antisense	ACCTTGACCCATGGCGCTGC
Luciferase reporter cloning	
proPEPCK-FW	GGTTCGCTAGCTGAGTTTGGTCGGAGG
proPEPCK-RV	CAGCAAGCTTGTGTTCCCAGTGGGAAG
proPEPCK-ROREmt-FW	CAGCAGCGCCATGGGTCAAGGTTTAGTCAG
proPEPEK-ROREmt-RV	CTGACTAAACCTTGACCCATGGCGCTGCTG
proPEPCK-C3mt-FW	GAAAAGGGTGTTGTGTTTCCGGACAGCAG
proPEPEK-C3mt-RV	CTGCTGTCCGGAAACACAACACCCTTTC
PGVB2-FW	CAAGTGCAGGTGCCAGAAC
PGVB2-RV	CACCTCGATATGTGCATCTG
qRT-PCR	
rtPEPCK-FW	AAGAAGTGCTTTGCTCTCAG
rtPEPCK-RV	GCCATTTTCTGGGTTGATGG
rtRORA-FW	TGTGATCGCAGCGATGAAAG
rtRORA-RV	ACAGGAGTAGGTGGCATTGC
rtACTB-FW	GCACCACACCTTCTACAATG
rtACTB-RV	CTCAAACATGATCTGGGTCATC
rt18SrRNA-FW	CGATAACGAACGAGACTCTGG
rt18SrRNA-RV	TAGGGTAGGCACACGCTGAGC
