# Supplementary material for: Phosphoenolpyruvate Carboxykinase, a Key Enzyme That Controls Blood Glucose, Is a Target of Retinoic Acid Receptor-Related Orphan Receptor α
Source: PLoS One. 2015 Sep 18;10(9):e0137955. doi: 10.1371/journal.pone.0137955 (PMC4575163; doi:10.1371/journal.pone.0137955)
Supplement: S2 Table — (PDF) [file pone.0137955.s002.pdf]

S2 Table. siRNAs. Synthesized siRNA sequence are underlined.

Primer	Sequence (5′–3′)
siGFP-sense-u	GATCACTAATACGACTCACTATAGGGCAAGCTGACCCTGAAGTTCTT
siGFP-sense-d	AAGAACTTCAGGGTCAGCTTGCCCTATAGTGAGTCGTATTAGTGATC
siGFP-antisense-u	GATCACTAATACGACTCACTATAGGGGAACTTCAGGGTCAGCTTGTT
siGFP-antisense-d	AACAAGCTGACCCTGAAGTTCCCCTATAGTGAGTCGTATTAGTGATC
siRORA258-sense-u	GATCACTAATACGACTCACTATAGGGGTCAGAAGAACTGTTTGATTT
siRORA258-sense-d	AAATCAAACAGTTCTTCTGACCCCTATAGTGAGTCGTATTAGTGATC
siRORA258-antisense-u	GATCACTAATACGACTCACTATAGGGATCAAACAGTTCTTCTGACTT
siRORA258-antisense-d	AAGTCAGAAGAACTGTTTGATCCCTATAGTGAGTCGTATTAGTGATC
siRORA1388-sense-u	GATCACTAATACGACTCACTATAGGGCTAATGGCATTTAAAGCAATT
siRORA1388-sense-d	AATTGCTTTAAATGCCATTAGCCCTATAGTGAGTCGTATTAGTGATC
siRORA1388-antisense-u	GATCACTAATACGACTCACTATAGGGTTGCTTTAAATGCCATTAGTT
siRORA1388-antisense-d	AACTAATGGCATTTAAAGCAACCCTATAGTGAGTCGTATTAGTGATC
siRORAmt-sense-u	GATCACTAATACGACTCACTATAGGGGATTACAACGAATCTAAGTTT
siRORAmt-sense-d	AAACTTAGATTCGTTGTAATCCCCTATAGTGAGTCGTATTAGTGATC
siRORAmt-antisense-u	GATCACTAATACGACTCACTATAGGGACTTAGATTCGTTGTAATCTT
siRORAmt-antisense-d	AAGATTACAACGAATCTAAGTCCCTATAGTGAGTCGTATTAGTGATC
